# Stable MXene Dough with Ultrahigh Solid Fraction and Excellent Redispersibility toward Efficient Solution Processing and Industrialization

**DOI:** 10.1002/advs.202300660

**Published:** 2023-04-20

**Authors:** Shungui Deng, Tiezhu Guo, Frank Nüesch, Jakob Heier, Chuanfang (John) Zhang

**Affiliations:** ^1^ College of Materials Science & Engineering Sichuan University Chengdu 610065 China; ^2^ Laboratory for Functional Polymers Swiss Federal Laboratories for Materials Science and Technology (EMPA) Überlandstrasse 129 Dübendorf CH‐8600 Switzerland; ^3^ Institute of Materials Science and Engineering Ecole Polytechnique Federale de Lausanne (EPFL) Station 12 Lausanne CH‐1015 Switzerland; ^4^ Key Laboratory of Multifunctional Materials and Structures Ministry of Education School of Electronic Science and Engineering Xi'an Jiaotong University Xi'an 710049 China

**Keywords:** dough, extrusion printing, inks, micro‐supercapacitors, transition metal carbides, two‐dimensional MXene

## Abstract

Two‐dimensional (2D) transition metal carbides, and/or nitrides, so‐called MXenes, have triggered intensive research interests in applications ranging from electrochemical energy storage to electronics devices. Producing these functional devices by printing necessitates to match the rheological properties of MXene dispersions to the requirements of various solution processing techniques. In particular, for additive manufacturing such as extrusion‐printing, MXene inks with high solid fraction are typically required, which is commonly achieved by tediously removing excessive free water (top‐down route). Here, the study reports on a bottom‐up route to reach a highly concentrated binary MXene‐water blend, so‐called MXene dough, by controlling the water admixture to freeze‐dried MXene flakes by exposure to water mist. The existence of a critical threshold of MXene solid content (≈60%), beyond which no dough is formed, or formed with compromised ductility is revealed. Such metallic MXene dough possesses high electrical conductivity, excellent oxidation stability, and can withstand a couple of months without apparent decay, providing that the MXene dough is properly stored at low‐temperature with suppressed dehydration environment. Solution processing of the MXene dough into a micro‐supercapacitor with gravimetric capacitance of 161.7 F g^−1^ is demonstrated. The impressive chemical and physical stability/redispersibility of MXene dough indicate its great promise in future commercialization.

## Graphical abstract

MXene dough with a high solid fraction up to 60 % is reported to exhibit non‐stick and reshapeable properties. The shelf‐life of MXene can be prolonged for couple of months with conductivity and redispersibility maintained in a practical storage method. The extruded MXene dough‐based micro‐supercapacitors show impressive charge storage performance, implying potential in electronic devices.

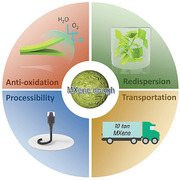

## 1 Introduction

MXene, as a rapid rising family of two‐dimensional (2D) material, has gained substantial interest due to its rich surface chemistry, extremely high electrical conductivity coupled with exceptional mechanical, electrochemical, and electromechanical properties.^[^ ^1^ ^]^ The general formula of MXene is M*_n_*_+1_X*_n_* (*n* = 1–4), which is synthesized by selective etching of element A from a M*_n_*_+1_AX*_n_* phase, where M is an early‐transition metal (Ti, Sc, Nb, V, Mo, Zr, Hf, Cr, etc.), A is Si, Al, Ga, etc., X is carbon or nitrogen.^[^ ^2^ ^]^ In addition, MXene can be readily delaminated into individual flakes by rigorously shaking or sonication.^[^ ^3^ ^]^ The hydrophilic terminations on the MXene surface endow homogeneous aqueous dispersions in the absence of any surfactants or polymeric additives, thus, enable the formulation of additive‐free MXene inks that further allows solution processing (including printing and coating) into various MXene‐based architectures at ease.^[^ ^4^ ^]^ By tuning the rheological properties of the MXene inks and matching the requirements of solution processing, one is capable to realize room‐temperature directing ink writing of various devices and patterns, such as sensors, micro‐supercapacitors (MSCs), micro‐batteries, actuators, antennas, radio‐frequency identification (RFID), etc.^[^ ^5^ ^]^ In particular, through elaborate surface engineering of the substrate, and/or tailoring the solvent chemistry of the inks, high‐precision, robust devices/patterns that strongly adhere to the substrates can be obtained, opening vast opportunities for printed flexible electronics in planted medical applications.^[^ ^6^ ^]^

The most popular solutions for adapting ink properties are tuning the concentration and/or selecting environmentally benign solvents with a relatively low boiling point.^[^ ^7^ ^]^ However, for MXene, solvent selection is more or less restricted to a few solvent where a homogeneous dispersion can be realized. Only those good solvents like ethanol, NMP, DMSO, etc. are able to disperse delaminated MXene nanosheets without quick sedimentation.^[^ ^8^ ^]^ On the other hand, tuning the ink concentration is more straightforward and efficient. For example, diluting the dispersion to a viscosity regime of 1–10 mPa s ensures stable jetting for inkjet printing of MXene inks, while concentrating the dispersion to a viscosity regime of 5–50 Pa s allows direct extrusion‐printing of MXene aqueous inks.^[^ ^9^ ^]^ It is worth mentioning that even at such a high viscosity range, the solid fraction (of delaminated MXene flakes) is still quite low, or the water content is very high. For instance, for MXene aqueous inks with concentration up to 100 mg mL^−1^, the MXene content is only 9 wt.% while the water content is 91 wt.%. While the high content of water (or other solvents) surges the cost for transporting a large amount of MXene samples/products, the more detrimental fact is that water oxidizes MXene flakes and deteriorates the inks in a short period of time. According to Zhang et al. and other reports,^[^ ^10^ ^]^ water is the main oxidant for the degradation of MXene aqueous inks. Despite replacing water with other polar solvents may work for elongating the MXene shelf life, other issues raise such as too low ink concentration, poor flake dispersibility, or hazardous chemicals.

As such, decreasing the water amount in aqueous MXene inks and recovering the homogeneous dispersion when necessary is of significance toward efficient solution processing and stable MXene storage. Although strategies focusing on the complete removal of water (such as freeze‐drying or vacuum‐filtrating, etc.) are able to yield freestanding MXene films/non‐stacked flakes or aerogels free from oxidation issues, it's quite challenging to perform sequential solution processing based on these fully dehydrated MXenes.^[^ ^11^ ^]^ This is due to the challenging, if not impossible, redispersion steps necessary to realize uniform inks consisting predominantly of single‐flakes. In other words, one needs to precisely control the water amount among MXene flakes such that the redispersibility and machinability of the resultant solids are maintained with minimum potential of oxidation.

Dough is a unique semi‐solid self‐standing material state containing a minimum amount of water, the latter provides the dough with cohesive, reshapable, and processable properties.^[^ ^12^ ^]^ Another important aspect is that highly concentrated MXene nanomaterials in the dough state allow high mass storage and efficient, cost‐effective transport of active materials. Synthesizing MXene dough is expected to greatly extend the shelf life of MXene, this is because 1) most free water and with it dissolved oxygen are removed in such a high MXene concentration state and 2) the residual water molecules are surrounded by MXene flakes, the proximity of the latter leading to a local confinement effect due to the steric shielding phenomenon.^[^ ^13^ ^]^ Moreover, the remaining amount of water allows the dough to be redispersed into concentrated homogeneous inks, with desirable viscoelastic properties, for subsequent solution processing such as extrusion printing. Liu et al. reported the synthesis of MXene dough by reducing the water amount in the MXene solutions through high‐speed centrifugation followed by direct drying.^[^ ^14^ ^]^ However, such a top‐down route involving cascading and heating hampers the precise control of the water content, causing uneven evaporation rates (thus leading to locally fully dried powders) as well as posing risks of partial MXene oxidation. In other words, straightforward formulation of high‐quality MXene dough featuring good redispersibility and long shelf life is of importance, but has proven to be quite challenging.

Herein, we report on the formulation of stable MXene dough through a bottom‐up strategy by adjusting the water content in freeze‐dried MXene flakes exposed to water mist. We reveal the critical threshold of 40 wt.% blended free water, beyond which the dough is no longer cohesive and shapable. Using the appropriate water admixture, such a MXene dough is able to be readily redispersed into homogenous MXene solutions featuring predominantly single‐layer MXene flakes. We demonstrate that, by suppressing the water evaporation rate, the MXene dough well maintains electrical conductivity, shapability, and dispersibilty after two months of storage in low‐temperature environment. We further show directly extruded all‐MXene‐based MSCs using MXene dough, indicating a high capacitance of 161.67 F g^−1^ per electrode. This work provides a route for stable long‐term storage of condensed MXene flakes, and may trigger promising applications in solution processing of high‐performance functional devices, ranging from microscale energy storage to sensing devices, to name just a few.

## 2 Results and Discussion

**Figure 1:**
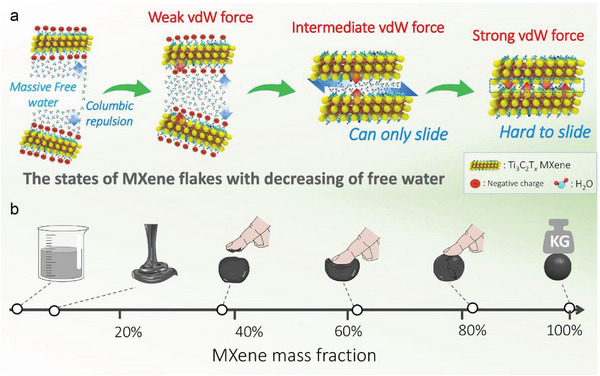
a) The interlayer interaction of MXene‐water mixture. b) Continuous mixture state transitions of binary MXene‐water blend with increasing of MXene mass fraction.

Unlike other 2D materials, MXene possess abundant hydrophilic, negatively‐charged terminal functional groups on the surface, endowing the direct formation of stable homogeneous colloidal aqueous solutions in the absence of any surfactants or polymers. In the MXene‐water binary system, the material state is MXene content‐dependent. For instance, in diluted MXene dispersion with ≈0.2 to 3.5 wt.% MXene (Figure S1a,b, Supporting Information), the excess free water suspends the as‐delaminated MXene flakes due to the low zeta potential (≈−40 mV).^[^ ^15^ ^]^ Increasing the MXene content (i.e., to 20 wt.%) results in the formation of binder‐free MXene viscous inks suitable for screen‐ or extrusion printing, as the storage modulus and the loss modulus are quite high (Figure S2, Supporting Information).^[^ ^9^ ^]^ The free water amount is also much reduced and confined within the interlayers (**Figure** **1**a). Further increasing the MXene content (i.e., to 40–60 wt.%) leads to a critical transition from sticky paste to non‐sticky, soft semi‐solids. In particular, the water molecules are able to slide between the delaminated MXene flakes, endowing good deformation responses upon external stimuli. We define the soft semi‐solids with shapable, cohesive and malleable properties as MXene dough at the critical threshold MXene content (≈60 wt.%), as shown in Figure S1c,f, Supporting Information.

It's worth noting that beyond this critical point, for example when the MXene content is above 80%, the proximity of nanosheets leads to strong steric shield effect, leading to the rapid increase of van der Waals (vdW) forces and hydrogen bonding between MXene interlayers while limiting the ability of the water molecules to slide between flakes.^[^ ^16^ ^]^ Consequently, the dough exhibits a hard solid state which is fragile and tends to be fractured into pieces upon external force, as shown in Figure 1b and Figure S1d,e, Supporting Information. The significant changes in the macroscopic properties of the MXene‐water binary system highlight the necessity of controlling the water content. Excessive free water results in inks or aqueous dispersions while low free water leads to hard, fragile solids. Therefore, to achieve cohesive, stable MXene dough with good ductile properties without oxidation or redispersibility issues, one needs to ensure the water content in the MXene‐water binary system falls in the range of 40–60 wt.%, as discussed below.

### 2.1 Synthesis and Properties of MXene Dough

**Figure 2:**
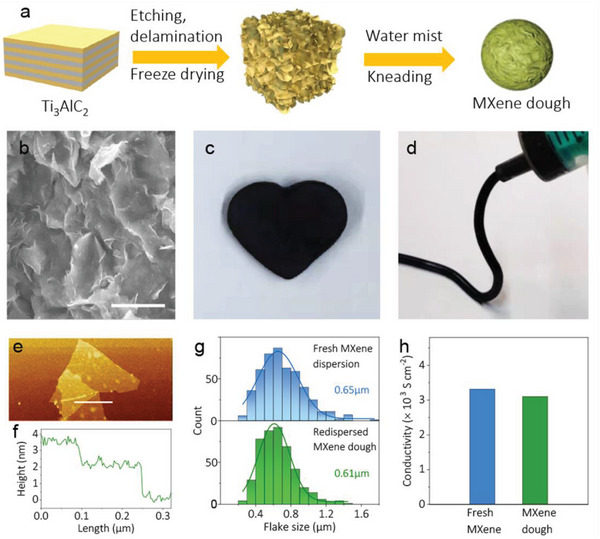
a) Schematic synthesis of MXene dough; b) SEM of the inner structure of MXene dough, scale bar is 20 µm; Photos for c) pinching and d) extrusion of MXene dough; e) AFM images and f) corresponding height profile along the line of redispersed suspended MXene from MXene dough; g) Lateral flake size distribution of a fresh MXene and redispersed MXene dispersion from MXene dough; h) The conductivity of a filtrated MXene film from fresh MXene and MXene dough, respectively.

We begin with the synthesis of MXene. A minimally intensive layer delamination (MILD) was employed to etch the precursor Ti_3_AlC_2_ MAX phase and subsequently to delaminate the multilayered Ti_3_C_2_T_x_ MXene through hand‐shaking and sonication.^[^ ^5^ ^b]^ The colloidal MXene dispersion was further subjected to freeze‐drying to obtain powders consisting of delaminated flakes (Figure S3a, Supporting Information). These dried flakes are ready to be wetted and welded by water droplets, a behavior that is similar to the welding effect in MXene films enabled by water droplets reported by Fan et al.^[^ ^17^ ^]^ By hydrating these delaminated flakes with water mist precisely controlled by a pressurized sprayer, one is able to obtain MXene dough through repeated kneading, as schematically demonstrated in **Figure** **2**a and Figure S3b‐d, Supporting Information. By controlling the amount of water mist compositing with freeze‐dried MXene flakes (water content of ≈40 wt.%), a soft, kneadable, and non‐stick MXene dough is obtained (tap density ≈2.8 g cm^−3^), as shown in Figure S4, Supporting Information.

According to the scanning electron microscopy (SEM) images, the as‐obtained MXene dough exhibits a compact ravine surface with flakes closely stacked on the surface of sphere (Figure S5a, Supporting Information), while the interior of the dough shows randomly oriented flakes, possessing short‐range order but long‐range disorder corresponding to isotropic behaviors (Figure 2b). The cross‐sectional morphology in the dough is illustrated in Figure S5b, Supporting Information. The uniformly distributed water molecules interact with MXene flakes via weak hydrogen bond, endowing MXene with good malleable, cohesive, and shapable properties. For example, the soft MXene dough can be molded into arbitrary shapes like a heart (Figure 2c), or continuously extruded into filaments without clogging issues (Figure 2d), suggesting promising applications in room temperature extrusion printing (or 3D printing) of high‐performance MSCs, sensors, antennas, etc.

The fresh MXene dough can be reversibly redispersed into homogeneous aqueous solution through manual shaking or brief sonication of the dough in water (Figure S6, Supporting Information). After redispersing, MXene dough is disentangled into monolayered flakes, with the flakes morphology and thickness quite similar to those of fresh MXene dispersions, as identified by SEM (Figure S7a, Supporting Information) and atomic force microscopy (AFM, Figure 2e,f; Figure S7b, c, Supporting Information). The lateral size histogram of the flakes in the redispersed sample changes only marginally compared to that of fresh MXene flakes, indicating that the redispersion process is highly reversible and free from fragmentation (Figure 2g). We note this as an important property as it maintains the high quality of MXene flakes, ensuring a reversible recovery to MXene inks with desired rheological properties while preserving the pristine electrical conductivity. For instance, the MXene films based on vacuum‐filtrated fresh MXene colloids and redispersed suspension (Figure S8, Supporting Information) possess a similar compact, wrinkled ravine surface, leading to quite close electrical conductivity (3103 S cm^−1^ for the redispersed sample versus 3316 S cm^−1^ for the pristine film, Figure 2h).

However, the redispersibility of MXene dough is water content‐dependent. Exposure to an open environment results in continuous evaporation of free water. For instance, only 12 wt.% was retained after keeping the dough for 32 h under ambient air, in sharp contrast to 58 wt.% in the fresh dough (Figure S9a, Supporting Information). X‐ray diffraction (XRD) patterns of MXene dough exposed for different durations to air are compared to that of fresh MXene dough as well as fresh freeze‐dried MXene powders. As shown in Figure S9b (Supporting Information), the characteristic peak – (002) plane of MXene downshifts to 5.6° in the fresh MXene dough from 6.8° in the fresh freeze‐dried MXene powders, indicating a much larger interlayer spacing due to the hydration of freeze‐dried flakes. The hydrated flakes gradually dehydrate by increasing the exposure time to ambient condition, and become a completely dehydrated dough after 15 days of exposure, in agreement with the (002) peak centering at exactly same degree as that of freeze‐dried flakes. The dehydration is also observed when MXene dough is stored under hermetic condition. As shown in Figure S10 (Supporting Information), after sealing the MXene dough in a hermetic vial over 2 months, the dough becomes fragile and less cohesive, and is posing a challenge for full recovery to a homogeneous solution. The hydration/dehydration phenomenon highlights the necessity of developing strategies for the stable storage of MXene dough, allowing quick redispersion into inks/dispersions when needed.

### 2.2 Stability Studies of MXene Dough

**Figure 3:**
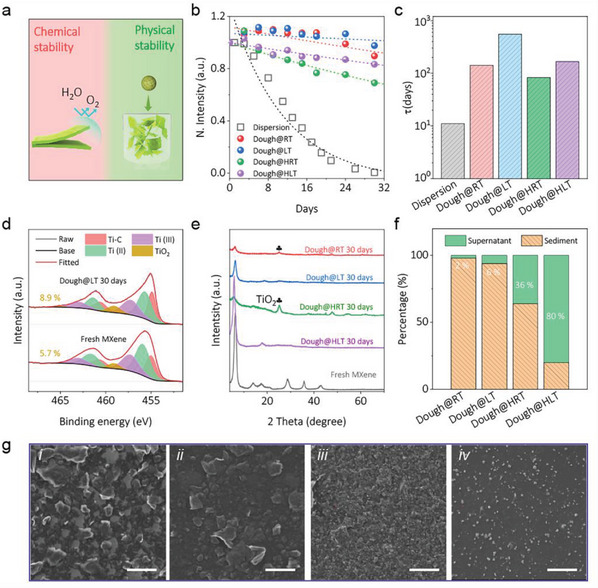
Stability of MXene dough in different storage conditions. a) Illustration of ideal storage with oxidation resistance and high redispersibility ; b) Normalized intensity degradation of the 785 nm characteristic peak of MXene dispersions from extinction UV‐vis spectra, the dotted lines are the fitting results according to the empirical equation *A* = *A_unre_* + *A_re_e* ^−*t*/*τ*^; c) Time constants of MXene dispersions in different environments; d) XPS of fresh MXene and Dough@LT stored for 30 days; e) XRD of MXene dough in different storage conditions for 30 days; f) The percentage of supernatant and sediment of MXene dispersions after above four 30‐days old doughs redispersed into water (manual shaking for 30 min) and kept at rest for over 30 hours; g) SEM to the MXene suspension after i) Dough@RT, ii) Dough@LT, iii) Dough@HRT and iv) Dough@HLT are stored over 30 days. The scale bars are 20 µm.

In principle, the ideal storage of MXene dough should ensure a minimum degree of oxidation and the maximum degree of redispersibility, as illustrated in **Figure** **3**a. As such, we evaluated the effects of storage temperature and humidity on these two essential parameters. As shown in Figure S11 (Supporting Information), four MXene doughs were stored at room temperature (Dough@RT), low temperature (Dough@LT), room temperature with humidity (Dough@HRT), and low temperature with humidity (Dough@HLT), respectively. By redispersing the dough and measuring the UV–vis spectra, one is able to monitor the oxidation extent on the MXene dough under different storage conditions. As indicated in Figure S12 (Supporting Information), the normalized extinction spectrum of pristine MXene dispersion showcases a rapid decay over time, best evidenced by the disappearance of the characteristic peak centering at 785 nm, a peak used for detecting the oxidation degree of MXene colloidal solutions. After 30 days, the peak intensity drops nearly to zero coupled with the formation of cloudy white dispersion, confirming the complete oxidation of MXene flakes (Figure S12a,b, Supporting Information). However, when processing the MXene dispersion into semi‐solid dough, the degradation phenomenon is much suppressed (Figure S12c‐f, Supporting Information). This is especially true when storing the MXene dough at low‐temperature, as intensity and position of the characteristic peak remain almost unchanged after 30 days of storage (Figure S12d, Supporting Information).

Figure 3b plots the normalized characteristic peak (at 785 nm) intensity of redispersed solution as a function of time after storing the dough in different environments. Also included are fitting curves according to the empirical law: A = *A_unre_* + *A_re_e* ^−*t*/*τ*^, where *A_unre_* and *A_re_* represent the unreactive and reactive MXene, respectively, and *τ* is the time constant (days). Generally, a larger *τ* suggests a longer storage stability against oxidation. The degradation kinetics follows the empirical law quite well, indicating the edge‐driven oxidation process which agrees with previous reports.^[^ ^10^ ^a,^ ^18^ ^]^ The fitting‐generated time constant *τ* for the different samples is compared in Figure 3c, showing a maximum stability in Dough@LT (556 days) which has increased by 50‐fold compared to that of MXene dispersion. This indicates that eliminating most free water and processing the dispersion into semi‐solid dough and storing under low‐temperature is quite effective in elongating the shelf life of MXenes. On the other hand, the direct exposure of MXene dough to external humidity greatly accelerates the oxidation (Dough@HRT, 83 days). X‐ray photoelectron spectra (XPS) shown in Figure 3d and Figure S13 (Supporting Information) also confirm the least oxidation (increased marginally to 8.9% of TiO_2_ from 5.7% in the fresh MXene) when storing the MXene dough at LT. In contrast, humidity apparently accelerates oxidation, as evidenced by the much‐increased content of TiO_2_ (15.7 %), as well as the formation of anatase in the X‐ray diffraction patterns (XRD, Figure 3e). The oxidation process is also suppressed by placing the dough in LT despite the direct exposure to external humidity, as the anatase peak is quite vague in the Dough@HLT (Figure 3e).

Besides oxidation resistance, the redispersibility of MXene dough is another important parameter to evaluate the stability. After storing in different environments over 30 days, the MXene doughs were redispersed into aqueous colloids through vigorous manual‐shaking. As shown in Figure S14 (Supporting Information), after being kept at rest for 30 hours, the supernatant of the colloid of Dough@RT group turns clear, while the other three groups are still black. The percentage of remaining suspended flakes were then evaluated by measuring the supernatant concentration, revealing only 2 % of MXene flakes in the colloidal suspension for Dough@RT, in contrast to 6%, 36%, and 80 % for Dough@LT, Dough@HRT, and Dough@HLT, respectively (Figure 3f). The maximum concentration in redispersed colloids for Dough@HLT suggests that humidity is beneficial for the improved redispersibility and physical stability of the MXene dough. The SEM images of redispersed MXene suspension from aged MXene doughs are shown in Figure 3g. The restacked MXene agglomerates and flake corners can be distinctly observed in Dough@RT and Dough@LT, which are absent in Dough@HRT and Dough@HLT. Instead, the latter two samples are decorated with abundant nanosized TiO_2_.

**Figure 4:**
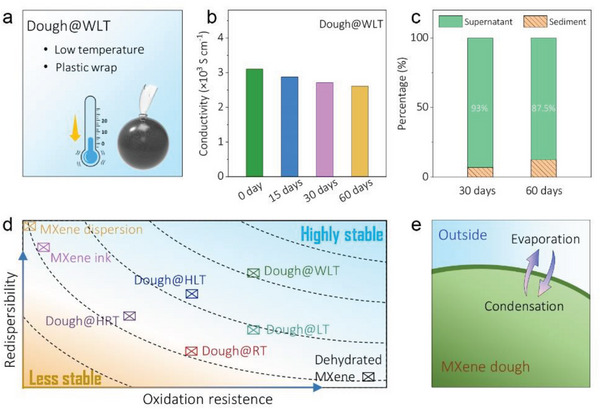
a) MXene dough is wrapped to seal and stored at room temperature; b) Conductivity change of Dough@WLT with days; c) The percentage of supernatant and sediment of MXene dispersions after 30‐days and 60‐days old Dough@WLT redispersed into water (manual shaking for 30 min) and kept at rest for over 30 hours; d) Scheme of oxidation resistance and redispersibility for different MXene state and different storage environment; e) Scheme of water dynamics in MXene dough.

The abovementioned results demonstrate that temperature and humidity greatly affect the oxidation stability and redispersibility/physical stability of MXene dough. Decreasing temperature helps the preservation of flakes while storing in the humid environment allows redispersing at ease. However, direct exposure to external humidity accelerates the oxidation process. As an alternative, suppressing the self‐evaporation kinetics of the fresh MXene dough through minimizing the free space above the dough in the vials is expected to maintain the pristine humidity, and thus preserves the hydration state. This is realized by tightly encapsulating the fresh MXene dough (60 wt.% of solid fraction) using clings film, and storing the dough at LT (labeling Dough@WLT), as shown in **Figure** **4**a.

Surprisingly, the conductivity of MXene has been well preserved, maintaining 2610 S cm^−1^ after 60 days of storage in Dough@WLT, in sharp contrast to 1070 S cm^−1^ after storing the dough in Dough@LT for 30 days (Figure 4b; Figure S15, Supporting Information). Filtrated films obtained from the redispersed colloids of aged Dough@WLT for 60 days also demonstrate rather similar surface morphology with that of fresh MXene dough (Figure S16, Supporting Information). In addition, the redispersed flakes of aged Dough@WLT for 30 days also show a smooth surface without either micro‐agglomerates or TiO_2_ nanoparticles (Figure S17, Supporting Information). Moreover, after 30 days and 60 days of storage in WLT, the percentage of redispersable and physically stable flakes reach 93 % and 87.5 %, respectively (Figure 4c; Figure S18, Supporting Information). These results demonstrate that maintaining the pristine hydration state through a simple tight encapsulation contributes to the excellent redispersibility of MXene dough.

### 2.3 Best Practice for Storing MXene Dough

With a view to stability concerns on MXene dough including chemical stability (that is, oxidation/degradation) and physical stability, with the latter essentially valuing the redispersibility of aged dough without sedimentation, one needs to suppress the oxidation/degradation kinetics of MXene dispersion to prolong the chemical stability of MXene dough. This can be done by introducing the steric shielding effect through concentrating the dispersion, forming MXene dough with a solid fraction above the critical threshold (40‐60 wt.%), as well as storing the dough at LT to suppress the oxidation kinetics.

Nevertheless, the physical stability/redispersibility of the MXene dough deteriorates with time, due to the dehydration of the dough which further leads to the irreversible formation of large agglomerates that cannot be further redispersed. Such decreased redispersibility with days can also be found in freeze‐dried MXene. As shown in Figure S19a (Supporting Information), unlike the excellent redispersibility of the fresh freeze‐dried MXene flakes, the 30 days‐old freeze‐dried MXene flakes are no longer redispersable through manual shaking, which can be attributed to the complete dehydration and formation of restacked agglomerates due to the strong vdW forces (Figure S19b, Supporting Information). It is worth mentioning that through appropriate sonication, these agglomerates can be redispersed at the cost of flake size and conductivity (Figure S20–S22, Supporting Information). Despite the direct exposure to external humidity is beneficial in boosting the dough redispersibility, humidity drives the degradation of the flakes. On the other hand, by elaborately maintaining the internal humidity through tightly sealing the dough, the dehydration kinetics is greatly suppressed, leading to much improved physical stability even after couple of months in WLT.

As such, we propose the best practice for long shelf‐life MXene dough by plotting the redispersibility and oxidation resistance, as schematically demonstrated in Figure 4d. While LT environment and freeze‐drying are good for chemical stability, the physical stability after redispersing MXene dough is poor. Combining LT with external humidity improves the physical stability with compromised chemical stability. On the other hand, through tight encapsulation and thus suppressing the water evaporation, dough dehydration kinetics is deaccelerated, maintaining the condensation on the surface. In addition, by controlling the relative humidity with different saturated metal salts in dough storage, it is also concluded that the partial water vapor pressure in the environment influences the MXene oxidation process (Figures S23 and S24, Supporting Information). Indeed, the equilibrium of water evaporation and condensation on the dough surface is important as it can be associated with the dough oxidation and MXene agglomerate formation (Figure 4e). Clarifying the mechanism of water on promoting the dough stability may need in‐depth investigation, but this is beyond the scope of this work.

### 2.4 Energy Storage Evaluation in all‐MXene Dough Micro‐Supercapacitors

**Figure 5:**
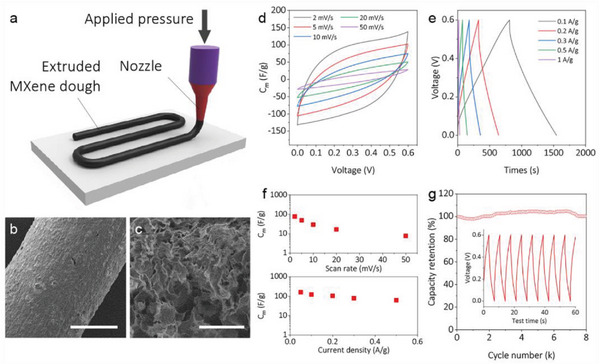
a) Schematic illustration of direct extrusion printing of MXene dough. SEM of b) an extruded MXene line and c) internal structure of extruded lines. The scale bars are 1 mm and 50 µm, respectively. d) Normalized cyclic voltammogram (CV) and e) galvanostatic charge‐discharge (GCD) curves of a typical MSC fabricated by extruded lines. f) Gravimetric capacitance (C*_m_*) of fabricated MSC obtained via CV and GCD. g) Long‐term cycling of the fabricated MSC with typical GCD curve inserted.

To demonstrate the potential application of stable MXene dough, we further directly extruded the dough into MSC patterns and evaluated the energy storage properties of the semi‐solid‐state devices. As shown in **Figure** **5**a and Figure S25a (Supporting Information), the soft MXene dough is directly extruded into cylindrical MXene lines with a diameter of 2 mm. Benefiting from the ultrahigh solid fraction of MXene dough, the extruded lines maintain the solid shape without spreading. After freeze‐drying, the solid MXene line is composed of densely stacked MXene on the surface and three‐dimensional porous structures in bulk (Figure 5b,c, Figures S26 and S27, Supporting Information) with a high mechanical strength. These lines can be stitched into various architectures such as, e.g., symmetric interdigitated structures (Figure S25b,c, Supporting Information). The specific capacitances of the extruded lines are first evaluated in a traditional three‐electrode configuration with H_2_SO_4_ as electrolyte (Figure S28, Supporting Information). The pseudo‐rectangular shape and slight redox peaks in the cyclic voltammograms (CV) demonstrate that the capacitance involves both Faradaic redox reaction and double‐layer capacitance. The specific capacitance is determined to be 279.8 F g^−1^ at 0.05 A g^−1^ according to the discharge behavior in galvanostatic charge‐discharge (GCD) curves. The behaviors of miniaturized supercapacitors (MSCs) are then demonstrated using these cylindrical lines as electrodes (Figure S29, Supporting Information) with PVA‐H_2_SO_4_ gel as electrolyte. The extruded MXene dough‐based MSC exhibits a capacitive charge‐storage behavior as verified by quasi‐rectangular CV and quasi‐linear, symmetric GCD curves (Figure 5d, e). The maximum specific capacitance reaches 77.48 F g^−1^ at 2 mV s^−1^ and 161.67 F g^−1^ at 0.05 A g^−1^ (per device), respectively, as indicated in Figure 5f. A satisfying cycling was also achieved within 8000 cycles with 100 % of initial capacitance retaining (Figure 5g). Since the active material loading of the 3D interdigitated device is as high as 0.14 g cm^−2^, the areal capacitance for each electrode can be calculated as ∼91 F cm^−2^ at the current density of 7 mA cm^−2^, which surpasses the capacitance performance of MXene‐based MSCs produced by other methods (Figure S30a, Supporting Information). The Ragone plot further showcases the potential of MXene dough‐based interdigitated electrode for high energy density supercapacitors, being orders of magnitude higher than other MSCs (Figure S30b, Supporting Information). At the current density of 7 mA cm^−2^, the calculated area energy density and power density are up to 1131.5 µWh cm^−2^ and 2100 µW cm^−2^, respectively. In addition, the tandem devices can easily power a bright light emitting diode (LED), demonstrating the practical application potential (Figure S31, Supporting Information).

To evaluate the charge‐storage capacities of the aged MXene dough, Dough@WLT was diluted into viscous and homogeneous inks. And all‐MXene‐based interdigitated MSC was produced using viscous MXene inks painted on A4 paper substrate (Figure S32a‐c, Supporting Information). A qualitative analysis through CV tests was carried out on MXene‐based MSCs with various MXene origin of fresh MXene, fresh MXene dough, and Dough@WLT stored for 60 days, respectively. Interestingly, the aged MXene dough‐based MSC showcases the best rate response compared to both of fresh MXene ink and MXene dough‐based MSC, as evidenced by the curves transited from "rugby ball" to quasi‐rectangular shape (Figure S32d‐f, Supporting Information). This is probably due to the slight oxidation during dough storage that promotes the redox reactions (Figure S33, Supporting Information).^[^ ^19^ ^]^ This important discovery indicates the great promise of our months‐old MXene dough for high‐performance miniaturized energy storage devices.

## 3 Conclusion

To conclude, we hydrated the freeze‐dried flakes using a controlled water mist, and synthesized MXene dough featuring excellent redispersibility and extremely high solid fraction up to 60 wt.%. The least amount of solvent in the dough and the redispersion at ease greatly reduce the transport cost while largely preserve the pristine properties of the flakes, therefore the MXene dough is of significance to the potential commercialization and academic collaborations. By adjusting the balance between dehydration and condensation kinetics via close wrapping using clings films, and storing the dough in LT environment, MXene dough with excellent oxidation stability and redispersibility/physical stability is achieved. Of equal importance is the direct extrusion of the MXene dough for the rapid production of quasi‐solid‐state MSCs, which can achieve a decent electrochemical charge storage performance. It is reasonable to believe that solution processing of MXene dough holds a bright future for the personal manufacturing of high‐performance electronic devices, such as micro‐batteries, sensors, antennas, etc. where conventional device fabrication is lengthy and energy‐consuming. The broad MXene family also provides vast opportunities for members with exotic properties to choose from.

## 4 Experimental Section

### Preparation of Ti_3_C_2_T_x_ MXene Dispersion

The preparation of delaminated Ti_3_C_2_T_x_ MXene was performed using the minimally intensive layer delamination (MILD) method. Typically, 2 g of MAX phase (Ti_3_AlC_2_, 11 technology, particle size ∼38 µm) was etched in a mixture of 40 mL 9 M of hydrochloric acid (HCl, 37 wt%, Sigma Aldrich, USA) and 3.2 g of lithium fluoride (LiF, Sigma Aldrich, USA) under vigorous stirring at 50 °C for 24 h. After etching, the suspension was transformed to centrifugal tubes and centrifuged and washed at 1500 rcf for at least 5 times till the supernatant is dark black, followed by vortex shaking for 30 min. Later, the suspension was sonicated for 1 h in an ice bath under Ar flow. Afterward, the suspension was centrifuged at 1500 rcf for 30 min and supernatant was then decanted. Finally, the supernatant was further centrifuged at 15 000 rcf for 30 min and the sediment was collected. MXene dispersion is obtained by adding water to the above sediment followed by vigorous shaking.

### Preparation of Freeze‐Dried MXene and MXene Dough

The freeze‐dried MXene flakes were prepared via freeze‐drying of delaminated colloids. Typically, the above MXene dispersion with a concentration of ≈5 mg mL^−1^ was prepared and then quickly frozen by immersion in a liquid nitrogen bath. After the MXene dispersion was completely frozen, it was then transferred to the freeze dryer with a vacuum of 0.05 mbar. A spongy freeze‐dried MXene was then formed after freeze‐drying for 2–3 days. MXene dough was obtained by hydrating the freeze‐dried MXene foam underwater mist, produced by a mist generator with pure water (Figure S2, Supporting Information). When the color of the foam darkened or was exposed to water mist for a certain of time, the hydrated MXene was kneaded and rolled into a sphere. The degree of hydration can be further tuned by adjusting the amount of additional water mist or freeze‐dried powders. When the dough does not leave extensive stains on gloves during kneading, the MXene mass fraction was around 60 wt. %.

### Materials Characterization

The delaminated MXene flakes were imaged by scanning electron microscopy (SEM, FEI Quanta 650) and atomic force microscopy (AFM, Nanoscope Icon 3, Bruker, Germany) techniques with nanoporous anodic aluminum Oxide (AAO) and silicon substrates, respectively. The SEM images of MXene agglomerates were obtained on silicon substrates. The SEM images of MXene dough were performed on the freeze‐dried MXene dough. The crystal structures of freeze‐dried MXene powder, water‐containing MXene doughs, and MXene films were characterized by X‐ray diffraction (XRD, BRUKER D8). X‐ray photoelectron spectroscopy (XPS, PHI Quantum 2000) was conducted to ascertain the valence states of Ti and calculate chemical content of TiO_2_ on the surface, where binding energy of sp^2^ C was set to 284.8 eV as standard. Anton Paar MCR 301 rheometer was used to test the rheology properties of MXene ink or MXene dough, with a parallel plate geometry of diameter of 25 mm and gap of 0.5 mm (rotational mode) and 2 mm (oscillatory mode). Thermogravimetric analysis (TGA) was carried out using a Perkin Elmer TGA7, using a heating rate of 12 °C min^−1^ and thereafter maintaining the temperature at 120 °C under a nitrogen gas flow. UV‐vis measurements were carried out on a UV–vis spectrophotometer (CARY 50 Scan) to the diluted MXene dispersion (≈0.007 mg mL^−1^). All the UV‐vis spectra were normalized to the local minimum at 220 nm, and the intensity at 785 nm was extracted as an indicator as a function of time.

### Conductivity Tests and Redispersibility Test

MXene conductivity is measured on infiltrated MXene films and calculated according to the following equation: *σ*_*DC*_ = 1/*R_s_d*, where *R_s_* and *d* is the sheet resistance and thickness of the film, respectively. Sheet resistance was measured using the four‐probe technique (JANDEL MODEL RM3‐AR). The thickness of filtrated film is measured by stylus profiler (DEKTAK 6 M). To test the redispersibility, MXene doughs were dispersed into water via handshaking for 30 min. The dispersion was then infiltrated into a film and dried, weighed as *m*_1_. After the dispersion is still over 30 h, same volume of supernatant was infiltrated into a film and dried, weighed as *m*_2_. The ratio of *m*_2_/*m*_1_ was used to evaluate the redispersibility of MXene dough.

### Fabrication of Three‐Electrode Configuration and MSC, and Electrochemical Measurements

The MXene dough was extruded into cylindrical MXene lines with 2 mm of diameter using a syringe and was straightened through the mold. The lines made from MXene dough were then frozen and freeze‐dried into solid lines. After freeze drying, the cylindrical shape as well as the diameter are maintained. The density of the freeze‐dried MXene line is weighed and calculated as ∼ 0.9 g cm^−3^. The electrochemical performance of the electrode was first measured in a three‐electrode configuration. The working electrode, counter electrode, and reference electrode consisted of a freeze‐dried MXene line, Pt electrode, and Ag/AgCl electrode, respectively, where H_2_SO_4_ (3 m) was used as electrolyte. The MSC module was then fabricated by pacing these lines (15 mm) as interdigitated electrodes on A4 paper with preset patterns, and counting the overlapped parts (10 mm) of the lines as electrode mass. The gap in interdigitated structure is 500 µm, and Ag silver paste is painted as current collector. The scheme is well illustrated in Figure S29 (Supporting Information). PVA‐H_2_SO_4_ gel electrolyte was then dropped onto the pattern area to completely wet the MSC electrode. PVA‐H_2_SO_4_ gel electrolyte is prepared as previously reported.^[^ ^20^ ^]^ Generally, 1 g PVA was dissolved in 10 mL H_2_O under constant stirring at 85 °C to obtain transparent solution and then cooled down to room temperature. Then, 3 g of H_2_SO_4_ (97 wt. %) was slowly added and further 1 h stirred to obtain PVA‐H_2_SO_4_ gel electrolyte.

The CV and GCD profiles was investigated on VSP potentiostat (BioLogic, France). The cyclic performances of MSC device were evaluated at 4 A g^−1^ for 8000 cycles on LAND battery testing system. The gravimetric capacitance C*_m_* (F g^−1^) was calculated by the following equation: *C*_*m*,*electrode*_ = *j*Δ*t*/*m_electrode_* Δ*V*, $C_{electrode}=\int_{0}^{0.6}jdV/m_{electrode}\Delta V\upsilon$, *C*_*m*, *cell*_ = *j*Δ*t*/*m_cell_* Δ*V*, $C_{m,\;cell}=\int_{0}^{0.6}jdV/m_{cell}\Delta V\upsilon$, where *j* is the discharge current (mA), Δ*t* is discharging time (s), *m* is electrode mass (g), Δ*V* is voltage window without voltage drop and υ is the scan rate (V s^−1^). For the Ragone plot, the areal energy density (E, µWh cm^−2^) and power density (P, µW cm^−2^) were calculated by the following equation: E = *C* × (Δ*V*)^2^/*A* × 2 × 3.6 , P = *E* × 3600/Δ*t* , where C is the area capacitance of the device (mF cm^−2^), A is total geometric area (cm^−2^), Δ*V* is discharge potential window and Δ*t* is discharging time.

## Conflict of Interest

The authors declare no conflict of interest.

## Supporting information

Supporting Information

## Data Availability

The data that support the findings of this study are available from the corresponding author upon reasonable request

## References

[1] a) Y. Gogotsi , B. Anasori , ACS Nano 2019, 13, 8491; 31454866 10.1021/acsnano.9b06394

[2] J. Halim , S. Kota , M. R. Lukatskaya , M. Naguib , M. Q. Zhao , E. J. Moon , J. Pitock , J. Nanda , S. J. May , Y. Gogotsi , M. W. Barsoum , Adv. Funct. Mater. 2016, 26, 3118.

[3] a) T. Z. Guo , M. S. Fu , D. Zhou , L. X. Pang , J. Z. Su , H. X. Lin , X. G. Yao , A. S. B. Sombra , Small Struct. 2021, 2, 2100015;

[4] a) M. Q. Zhao , C. E. Ren , Z. Ling , M. R. Lukatskaya , C. F. Zhang , K. L. Van Aken , M. W. Barsoum , Y. Gogotsi , Adv. Mater. 2015, 27, 339; 25405330 10.1002/adma.201404140

[5] a) B. Akuzum , K. Maleski , B. Anasori , P. Lelyukh , N. J. Alvarez , E. C. Kumbur , Y. Gogotsi , ACS Nano 2018, 12, 2685; 29463080 10.1021/acsnano.7b08889

[6] Y. Z. Shao , L. S. Wei , X. Y. Wu , C. M. Jiang , Y. Yao , B. Peng , H. Chen , J. T. Huangfu , Y. B. Ying , C. Zhang , J. F. Ping , Nat. Commun. 2022, 13, 3223. 35680851 10.1038/s41467-022-30648-2 PMC9184614

[7] C. Larsen , P. Lundberg , S. Tang , J. Rafols‐Ribe , A. Sandstrom , E. M. Lindh , J. Wang , L. Edman , Nat. Commun. 2021, 12, 4510. 34301943 10.1038/s41467-021-24761-x PMC8302666

[8] K. Maleski , V. N. Mochalin , Y. Gogotsi , Chem. Mater. 2017, 29, 1632.

[9] S. Abdolhosseinzadeh , X. T. Jiang , H. Zhang , J. S. Qiu , C. F. Zhang , Mater. Today 2021, 48, 214.

[10] a) C. F. J. Zhang , S. Pinilla , N. McEyoy , C. P. Cullen , B. Anasori , E. Long , S. H. Park , A. Seral‐Ascaso , A. Shmeliov , D. Krishnan , C. Morant , X. H. Liu , G. S. Duesberg , Y. Gogotsi , V. Nicolosi , Chem. Mater. 2017, 29, 4848;

[11] a) S. J. Wan , X. Li , Y. Chen , N. N. Liu , Y. Du , S. X. Dou , L. Jiang , Q. F. Cheng , Science 2021, 374, 96; 34591632 10.1126/science.abg2026

[12] a) C. N. Yeh , H. Y. Huang , A. T. O. Lim , R. H. Jhang , C. H. Chen , J. X. Huang , Nat. Commun. 2019, 10, 422; 30679461 10.1038/s41467-019-08389-6 PMC6345773

[13] a) M. Ghidiu , M. R. Lukatskaya , M. Q. Zhao , Y. Gogotsi , M. W. Barsoum , Nature 2014, 516, 78; 25470044 10.1038/nature13970

[14] Z. M. Fan , H. Y. He , J. X. Yu , J. F. Wang , L. Yin , Z. J. Cheng , Z. M. Xie , Y. S. Wang , Y. Y. Liu , ACS Mater. Lett. 2020, 2, 1598.

[15] K. A. S. Usman , S. Qin , L. C. Henderson , J. Z. Zhang , D. Y. Hegh , J. M. Razal , Mater. Horiz. 2021, 8, 2886. 34724521 10.1039/d1mh00968k

[16] E. Marquis , M. Cutini , B. Anasori , A. Rosenkranz , M. C. Righi , ACS Appl. Nano Mater. 2022, 5, 10516. 36062064 10.1021/acsanm.2c01847 PMC9425433

[17] J. F. Wang , Y. Y. Liu , Y. Q. Yang , J. Q. Wang , H. Kang , H. P. Yang , D. J. Zhang , Z. J. Cheng , Z. M. Xie , H. F. Tan , Z. M. Fan , Matter 2022, 5, 1042.

[18] D. Hanlon , C. Backes , E. Doherty , C. S. Cucinotta , N. C. Berner , C. Boland , K. Lee , A. Harvey , P. Lynch , Z. Gholamvand , S. F. Zhang , K. P. Wang , G. Moynihan , A. Pokle , Q. M. Ramasse , N. McEvoy , W. J. Blau , J. Wang , G. Abellan , F. Hauke , A. Hirsch , S. Sanvito , D. D. O'Regan , G. S. Duesberg , V. Nicolosi , J. N. Coleman , Nat. Commun. 2015, 6, 8563. 26469634 10.1038/ncomms9563 PMC4634220

[19] a) J. Tang , T. S. Mathis , N. Kurra , A. Sarycheva , X. Xiao , M. N. Hedhili , Q. Jiang , H. N. Alshareef , B. M. Xu , F. Pan , Y. Gogotsi , Angew Chem Int Ed Engl 2019, 58, 17849; 31574196 10.1002/anie.201911604

[20] C. F. Zhang , M. P. Kremer , A. Seral‐Ascaso , S. H. Park , N. McEvoy , B. Anasori , Y. Gogotsi , V. Nicolosi , Adv. Funct. Mater. 2018, 28, 1705506.

