# Supplementary material for: Stable MXene Dough with Ultrahigh Solid Fraction and Excellent Redispersibility toward Efficient Solution Processing and Industrialization
Source: Adv Sci (Weinh). 2023 Apr 20;10(19):2300660. doi: 10.1002/advs.202300660 (PMC10323650; doi:10.1002/advs.202300660)
Supplement: Supplementary file 1 — Supporting Information [file ADVS-10-2300660-s001.pdf]

ADVANCED SCIENCE
Open Access

Supporting Information

for *Adv. Sci.*, DOI 10.1002/advs.202300660

Stable MXene Dough with Ultrahigh Solid Fraction and Excellent Redispersibility toward Efficient Solution Processing and Industrialization

Shungui Deng, Tiezhu Guo, Frank Nüesch, Jakob Heier and Chuanfang (John) Zhang**

Supporting Information

Stable MXene Dough with Ultrahigh Solid Fraction and Excellent Redispersibility Toward Efficient Solution Processing and Industrialization

Shungui Deng[1,2,3], *Tiezhu Guo*[2,4], *Frank Nüesch*[2,3], *Jakob Heier*[2,*], *and Chuanfang (John) Zhang*[1,*]

[1] College of Materials Science & Engineering, Sichuan University, 610065 Chengdu, China

[2] Laboratory for Functional Polymers, Swiss Federal Laboratories for Materials Science and Technology (EMPA), Überlandstrasse 129, CH-8600 Zürich, Switzerland

[3] Institute of Materials Science and Engineering, Ecole Polytechnique Federale de Lausanne (EPFL), Station 12, CH-1015 Lausanne, Switzerland

[4] Key Laboratory of Multifunctional Materials and Structures, Ministry of Education, School of Electronic Science and Engineering, Xi'an Jiaotong University, Xi'an 710049, China

Corresponding author: chuanfang.zhang@scu.edu.cn, Jakob.heier@empa.ch

Keywords: Two-dimensional MXenes, transition metal carbides, inks, dough, extrusion printing, micro-supercapacitors

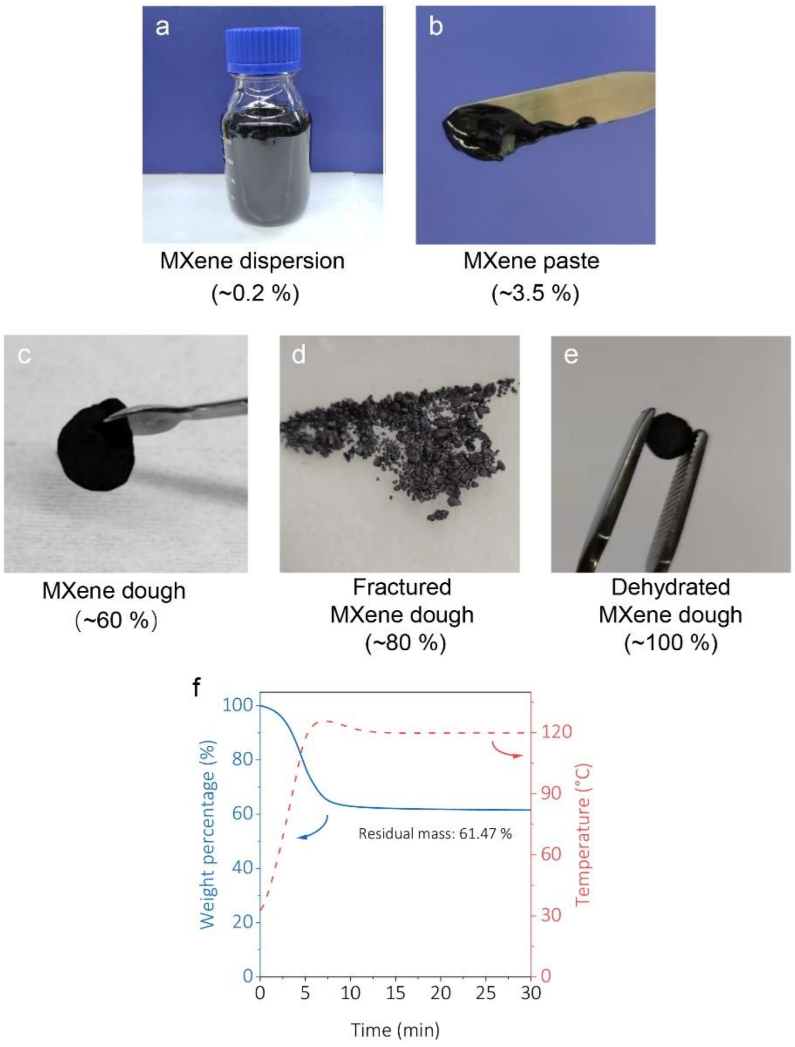

Supplementary Figure 1. Photos showing a binary MXene-water blend transitioning from **a**) dispersion, **b**) paste, **c**) melleable dough, **d**) fractured MXene dough to **e**) dehydrated MXene dough. f) TGA to kneadable and non-stick MXene dough, the result confirms the ~60 % MXene content as critical threshold.

MXene dough with non-sticky and good deformation properties exhibit a MXene mass fraction of ~60 %, beyond which the dough will be less ductile. When the MXene fraction is further increased to 80 %, it is difficult to moisten the MXene flakes uniformly and thus the dough loses the continuity and deformative properties. Further pressing and trying to knead the dough would cause the dough to be easily disintegrated into many small and hard fractures

that cannot be reconstituted. When MXene dough is completely dried, the dough become hard and cannot be shaped anymore.

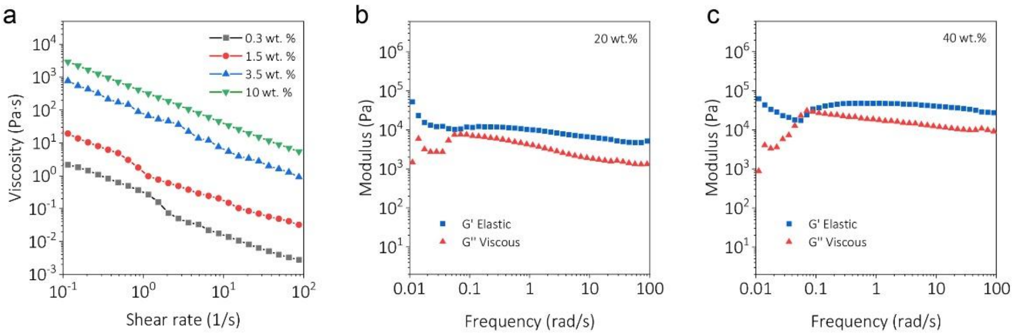

Supplementary Figure 2. **a**) Viscosities of $Ti_3C_2T_x$ MXene-water blends with various concentrations and viscoelastic properties of MXene-water mixtures with MXene mass fraction of **b**) 20 wt. % and **c**) 40 wt. %.

A simple shear experiment has been conducted on $Ti_3C_2T_x$-water blend to assess the viscous properties at various MXene contents as shown in Figure a. Results show that the MXene inks demonstrate shear shinning behavior, viscosities vary over orders of magnitude depending on concentration. Further increasing the MXene content (20 wt. %) results in the high storage modulus and loss modulus that higher than 1000 Pa, which offer processability for the fabrication techniques that require higher elastic modulus such as extrusion printing.

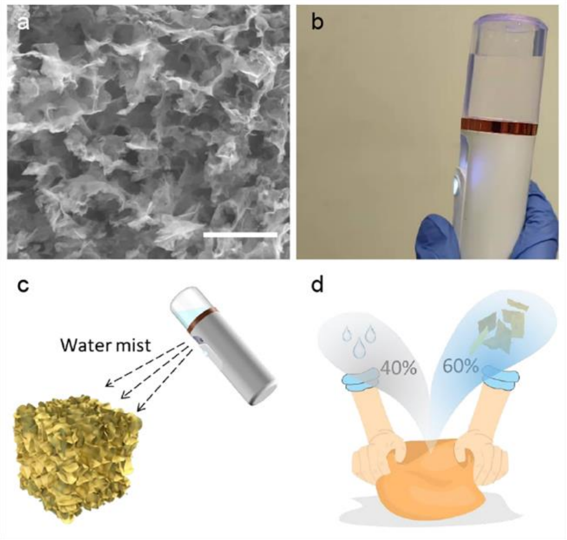

Supplementary Figure 3. a) SEM of freeze-dried MXene, scale bar is 20 μm. **b**) Photo of mist generation. **c-d**) Diagram of MXene dough preparation by hydrating freeze-dried MXene by spraying water mist and kneading.

Since the apparent volume of freeze-dried MXene is much larger than the volume of water needed, adding a small amount of water droplet usually results in a non-uniform hydration of MXene powders, where small aliquots of water tend to be absorbed locally. In order to obtain uniform MXene-water mixtures, aerosolized water mists were applied to hydrate the fresh freeze-dried MXene. MXene dough was obtained by further kneading and rolling of the hydrous foams.

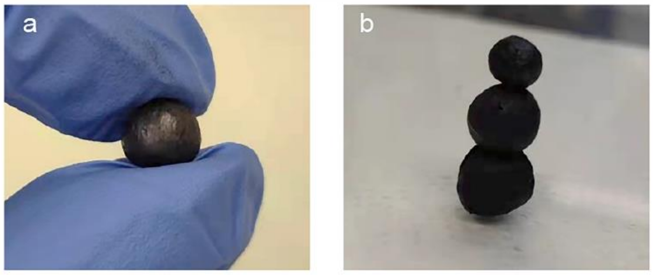

Supplementary Figure 4. Photos showing MXene with **a**) non-stick and **b**) cohesive property.

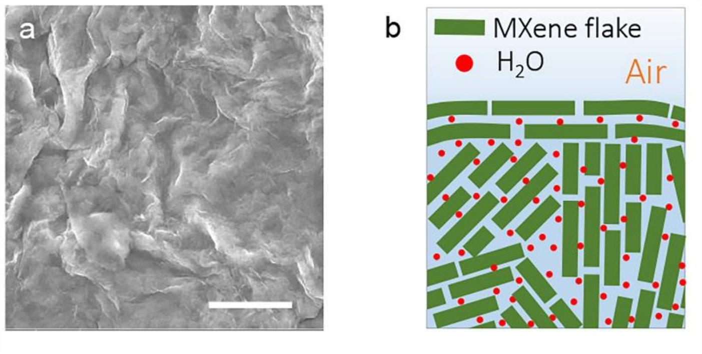

Supplementary Figure 5. a) Morphology of the surface of MXene dough, scale bar is 20 μm. **b**) Schematic illustration of the cross-section structure of MXene dough.

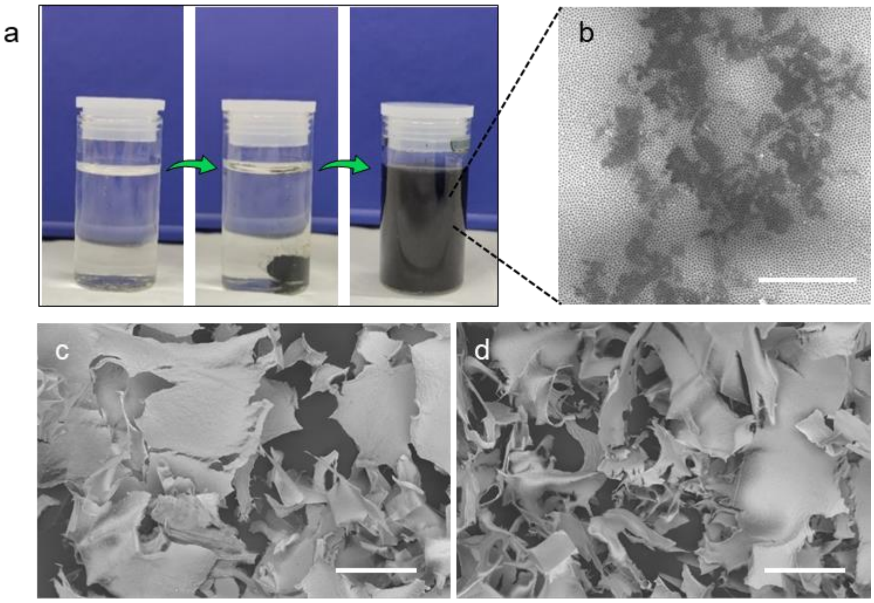

Supplementary Figure 6. a) MXene dough is readily redispersed into water and **b**) SEM of corresponding MXene dispersion, scale bar is 3 μm. SEM of fresh freeze-dried MXene powder from **c**) fresh MXene solution and **d**) dispersed MXene dough. The scale bar is 200 μm and the concentration of MXene dispersion is 2 mg/mL. The MXene structure of redispersed MXene dispersion is identical to the initial freeze-dried MXene due to the high redispersibility of MXene dough.

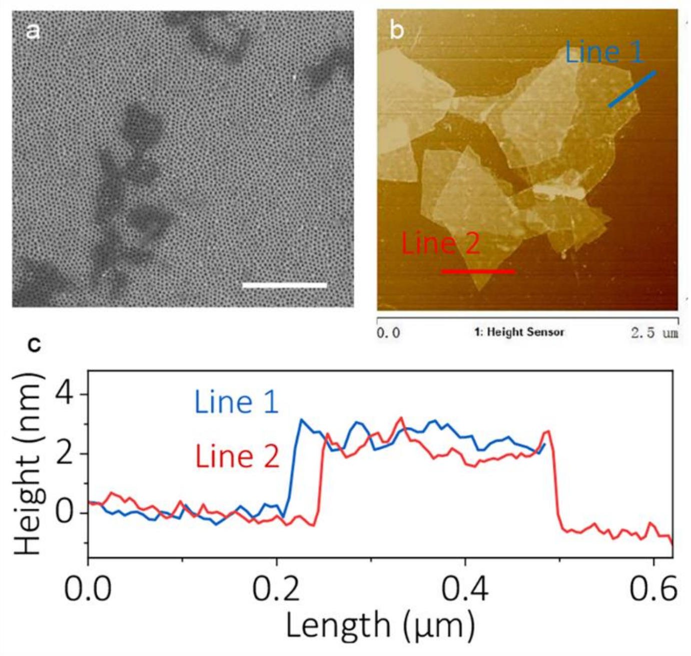

Supplementary Figure 7. a) SEM, **b**) AFM image of fresh MXene flakes and **c**) the corresponding height profile along the lines. The scale bar in SEM is 2 μm.

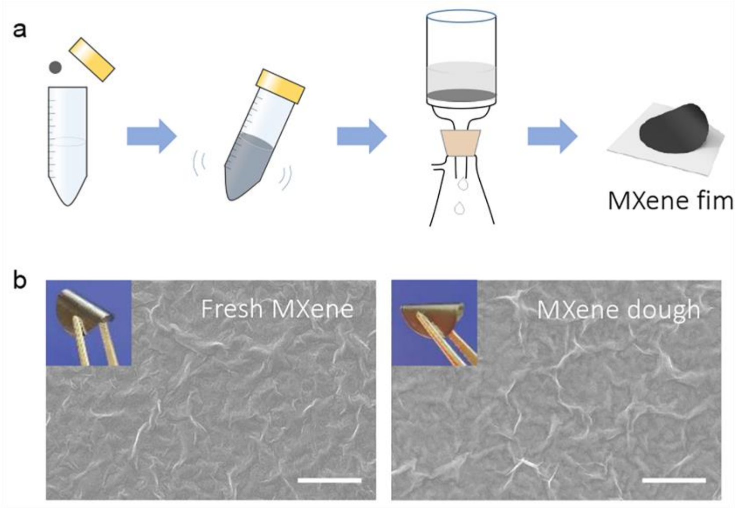

Supplementary Figure 8. a) Schematics of filtrated MXene film preparation from MXene dough; **b**) The surface morphology of filtrated MXene film from fresh MXene and MXene dough, respectively.

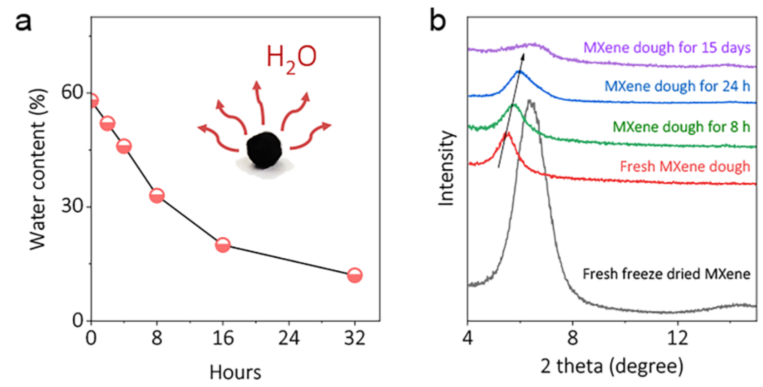

Supplementary Figure 9. a) Continuous evaporation when exposing MXene dough in the air, with the water content decreasing, the diameter of MXene dough is 3.5 mm. **b**) XRD of fresh freeze-dried MXene and MXene dough exposing for different durations in the air.

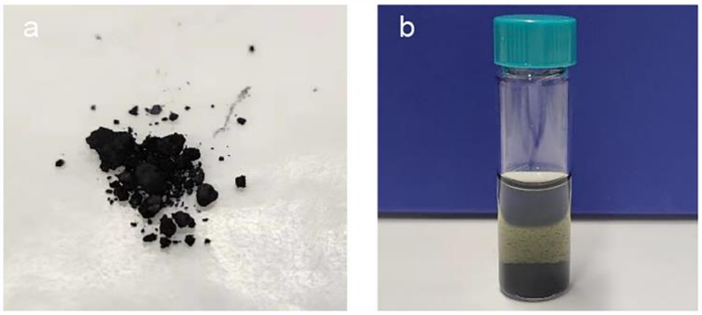

Supplementary Figure 10. a) The fragile MXene dough and **b**) the dispersion in water via handshaking after MXene dough is hermetically stored for 2 month.

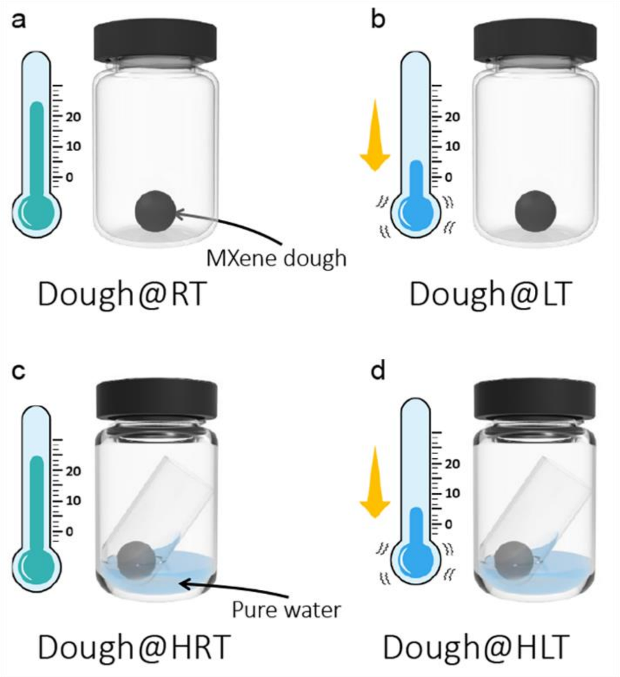

Supplementary Figure 11. Comparison groups of different storage environment for MXene doughs. MXene doughs were hermetically stored in the vials with different storage environments of **a**) Room temperature (denoted Dough@RT); **b**) Low temperature (denoted Dough@LT); **c**) Humid environment and room temperature (denoted Dough@HRT); **d**) Humid environment and low temperature (denoted Dough@HLT).

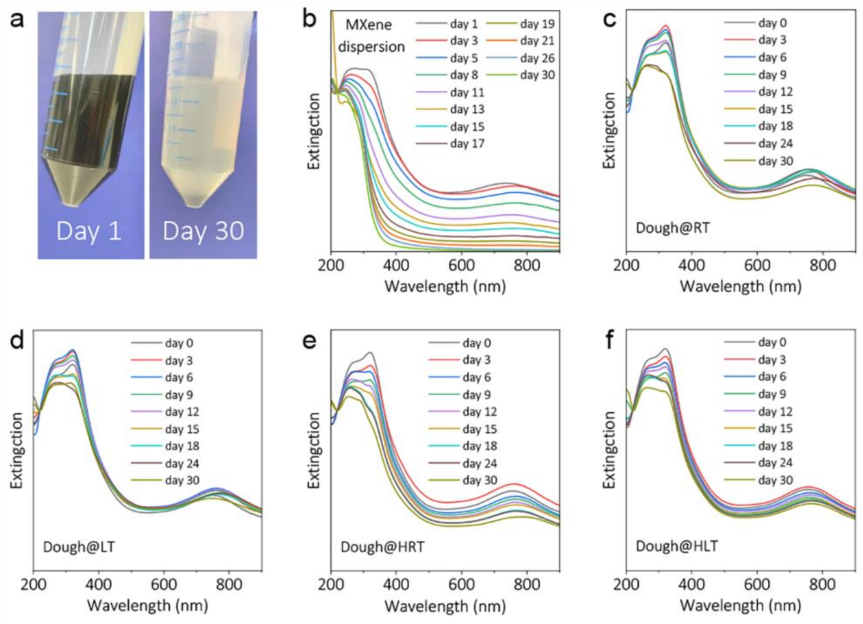

Supplementary Figure 12. a) The photos of MXene dispersion of day 1 and day 30; **b**) Normalized UV-vis extinction spectra of MXene dispersion; The normalized UV-vis extinction spectra of redispersed MXene dispersion from **c**) Dough@RT, **d**) Dough@LT, **e**) Dough@HRT and **f**) Dough@HLT.

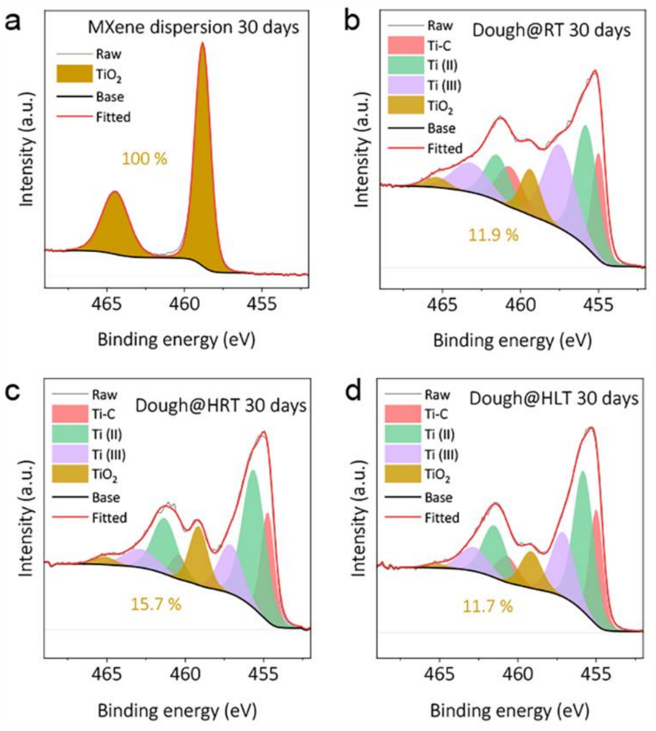

Supplementary Figure 13. XPS of **a**) MXene dispersion stored for 30 days, where we can find that the $Ti_3C_2T_x$ MXene is completely oxidized to TiO_2; **b**) Dough@RT stored for 30 days; **c**) Dough@HRT stored for 30 days and **d**) Dough@HLT stored for 30 days.

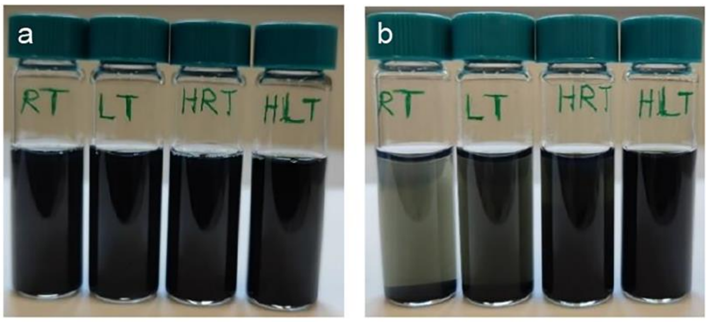

Supplementary Figure 14. Photos of **a**) redispersed MXene dispersion of MXene dough at different storage environment and **b**) after standing still over 30 hours. The redispersed MXene concentration is 2 mg/mL.

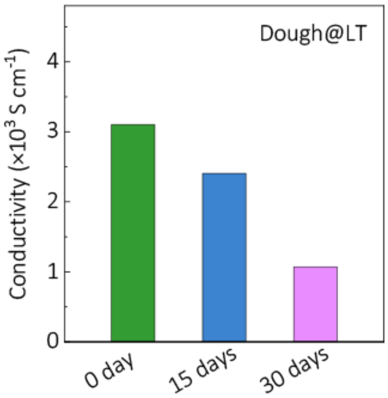

Supplementary Figure 15. Conductivity change of Dough@LT with days.

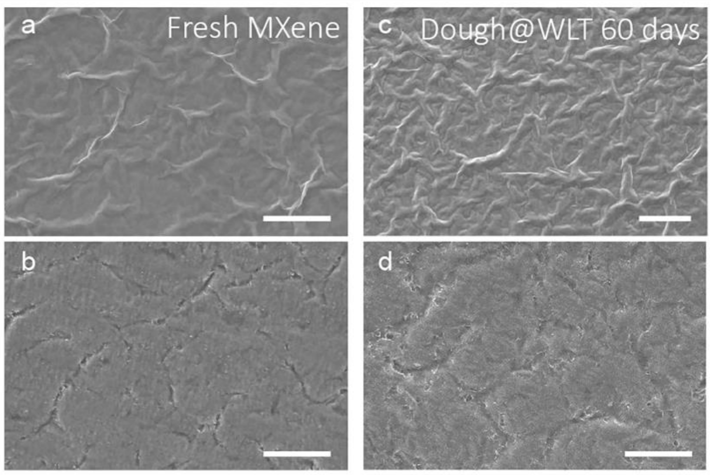

Supplementary Figure 16. The morphology of **a**) upside, **b**) downside of filtrated film from fresh Mxene dough and **c**) upside, **d**) downside of filtrated film from Dough@WLT storing for 60 days. The scale bars are 10 μm, 10 μm, 25 μm and10 μm, respectively.

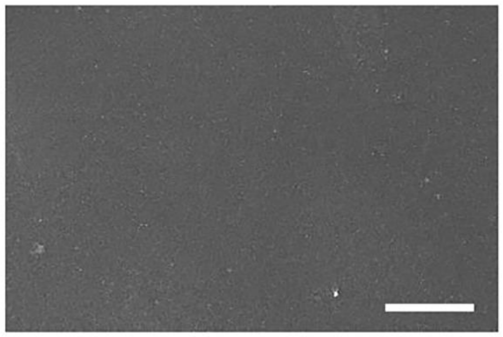

Supplementary Figure 17. SEM to the MXene suspension after Dough@WLT are stored over 30 days, scale bar is 25 μm.

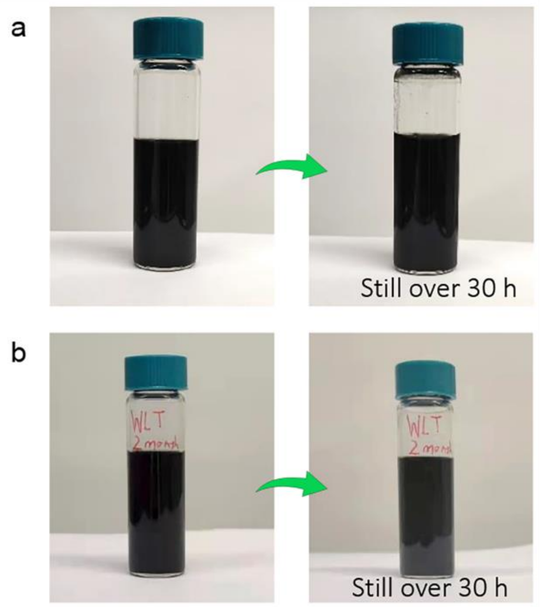

Supplementary Figure 18. Photograph of dough@WLT stored for **a**) 30 days and **b**) 60 days redispersed into water and standing still over 30 hours.

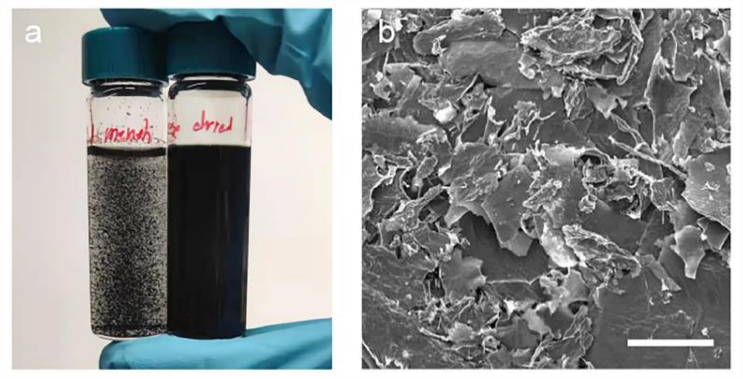

Supplementary Figure 19. **a**) Photo of the same amount of freeze-dried MXene on the first day (right) and stored for 30 days (left, stored at room temperature) dispersing into water, respectively; **b**) SEM of the undispersed freeze-dried MXene, scale bar is 100 μm.

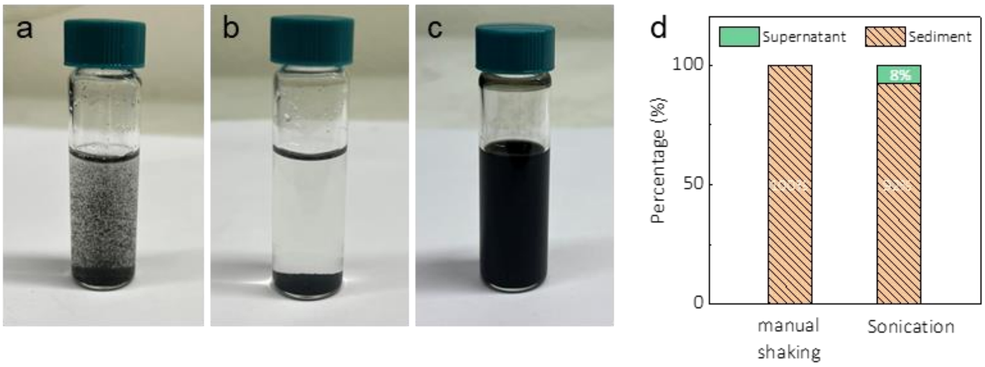

Supplementary Figure 20. Photos of **a**) dispersion of 10 mg freeze-dried MXene stored for 30 days dispersing into water. Photos of above dispersion **b**) manually shaken or **c**) sonicated for 30 min and then kept at rest for 30 hours. **d**) The percentage of supernatant and sediment of 30-days aged freeze-dried MXene dispersed into water (manual shaking or sonication for 30 min then keeping at rest for 30 hours).

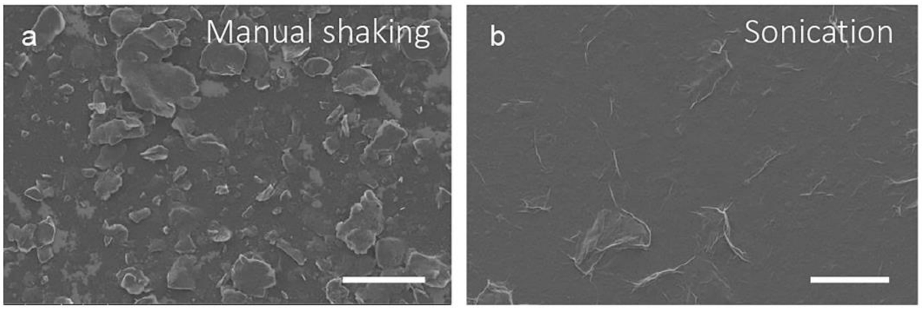

Supplementary Figure 21. SEM of redispersed MXene with **a**) manual shacking and **b**) sonication for 30 min of Dough@LT stored over 60 days. The scale bars are 30μm.

The formation of MXene agglomerate is the main reason to the limited redispersibility, as the restacked MXene cannot be redispersed by manual shaking. However, sonication process can further delaminate the restacked MXene agglomerate. But the high-energy sonication can also bring negative effects like the decrease of flake size and conductivity.[1]

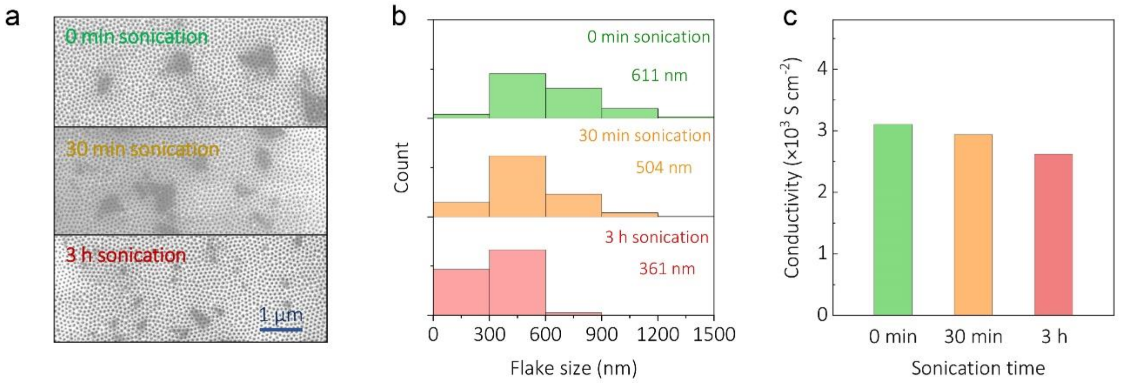

Supplementary Figure 22. Redispersion of MXene dough into water. **a**) SEM of MXene flakes, **b**) lateral flake size distribution, **c**) conductivities of the filtrated films with 0 min sonication, 30 min sonication and 3 h sonication time. This experiment supported that sonication treatment to MXene dispersion will bring about negative impacts on the quality of MXenes by decreasing their size and conductivity.

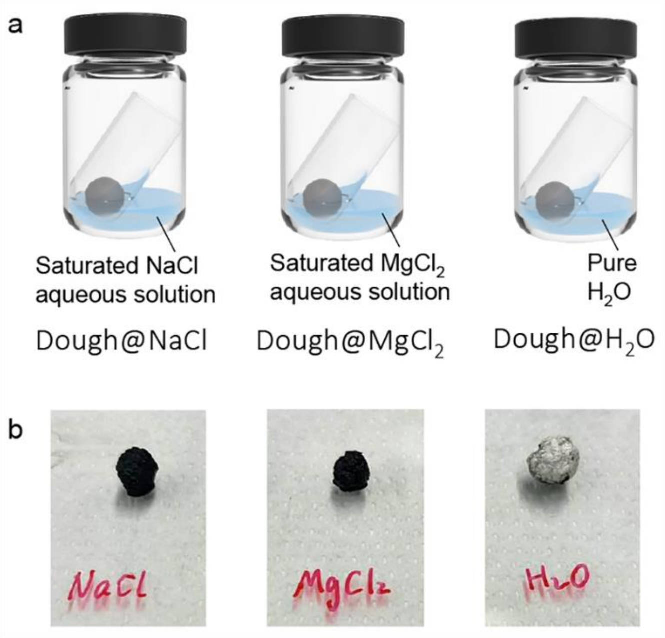

Supplementary Figure 23. a) MXene doughs were hermetically stored in the vials with different water vapor pressure. **b**) The photos of MXene dough after stored for 6 months at above environments.

The MXene doughs stored under different humidity (different water pressure) was explored. The saturated NaCl, saturated $MgCl_2$ and pure water is known to control the relative humidity in the hermetical bottle as 75.5 %, 32.5 % and 100 %, respectively.[2] After being stored for 6 month, we find that the Dough@NaCl and Dough@$MgCl_2$ were dehydrated, while Dough@H_2O was further wetted. We also find the surface of Dough@H_2O turned white, indicating the heavy oxidation.

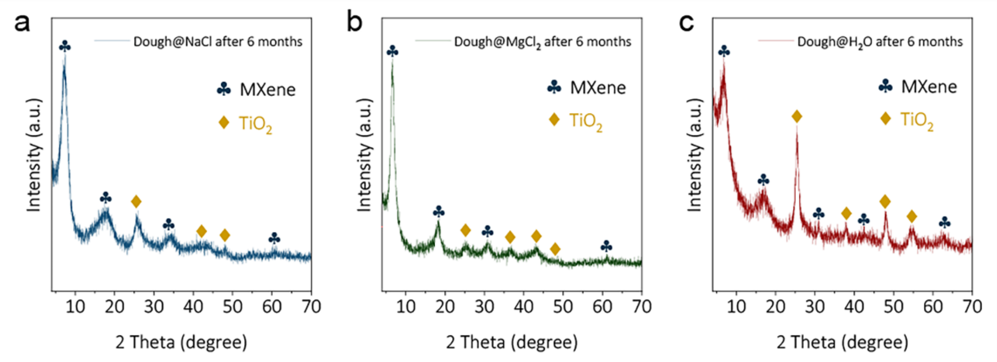

Supplementary Figure 24. The XRD of **a**) Dough@NaCl, **b**) Dough@$MgCl_2$, **c**) Dough@Air and **d**) Dough@H_2O stored for 6 months.

The Dough@NaCl, Dough@$MgCl_2$ and Dough@H_2O stored for 6 month were further dispersed in water via sonication and then filtrated into films. The XRD results verified the oxidation is suppressed after decreasing the water pressure as evidenced by the decreased TiO_2 peak intensity at the degree of 2θ= 25°. Such experiment supports the partial water vapor pressure in the environment influences the MXene oxidation process.

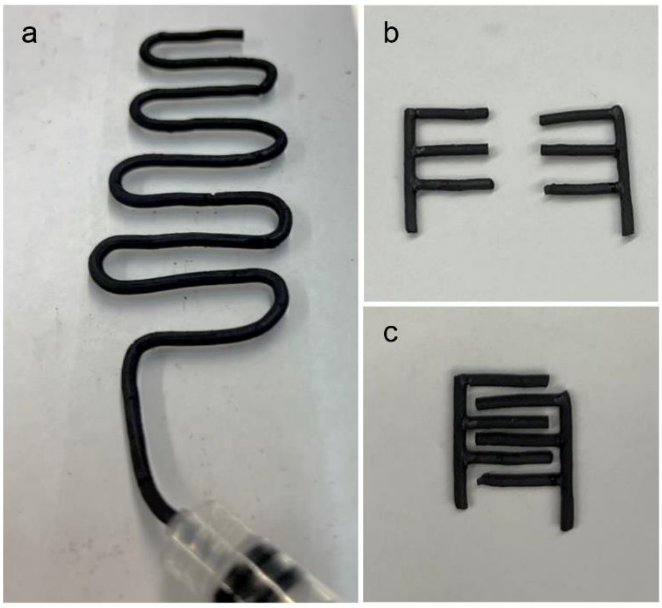

Supplementary Figure 25. a) The extruded MXene line from $Ti_3C_2T_x$ MXene dough. **b, c**) The freeze-dried MXene lines can be integrated as architectures, the lines are glued by MXene paste.

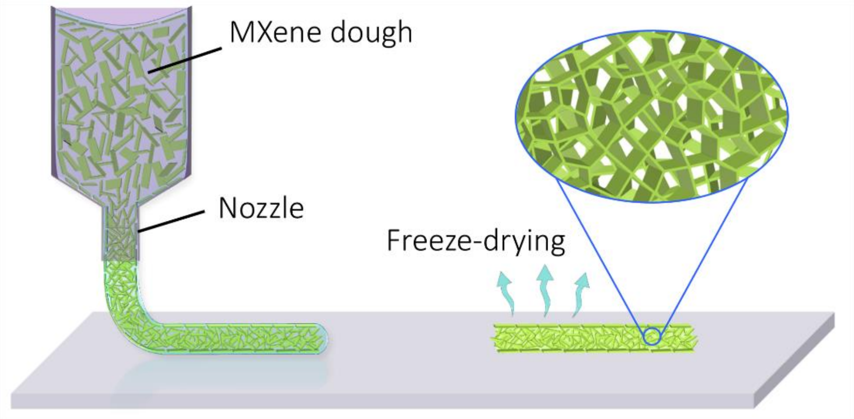

Supplementary Figure 26. Schematic diagram showing the preparation of the extruded MXene line from $Ti_3C_2T_x$ MXene dough.

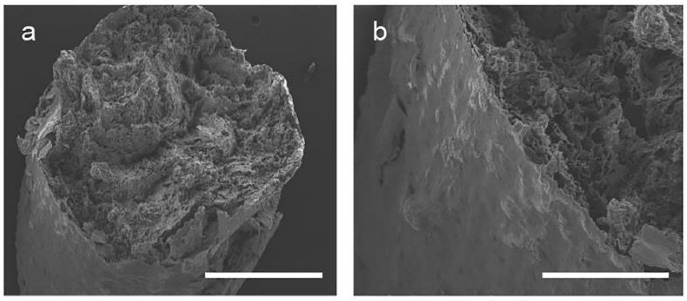

Supplementary Figure 27. a, b) SEM of cross-section morphology of freeze-dried MXene lines from MXene dough. Scale bars are 1 mm and 300 µm, respectively.

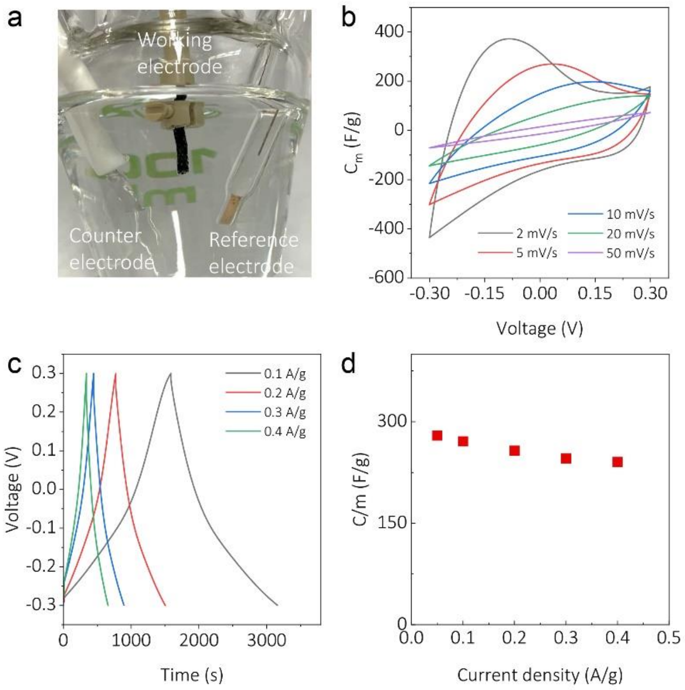

Supplementary Figure 28. a) Photograph of the three-electrode configuration, where working electrode is printed MXene lines, counter and reference electrode are Pt and Ag/Ag electrode, respectively. Electrochemical performance of **b**) CV, **c**) GCD and **d**) specific capacitances at different current densities.

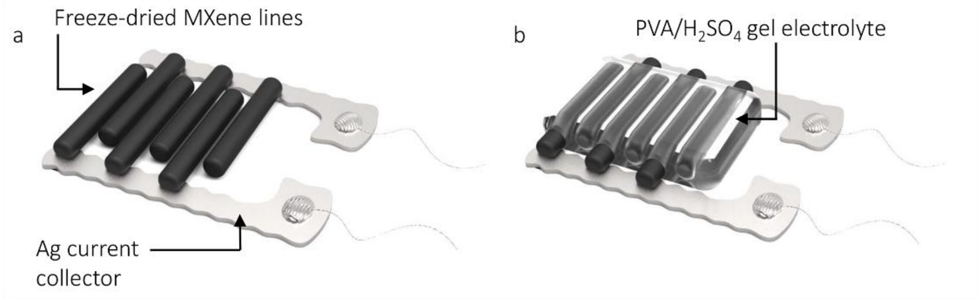

Supplementary Figure 29. a) Scheme of the fabrication of MSC by using freeze-dried cylindrical MXene lines and **b**) MSC with PVA/H_2SO_4 gel electrolyte.

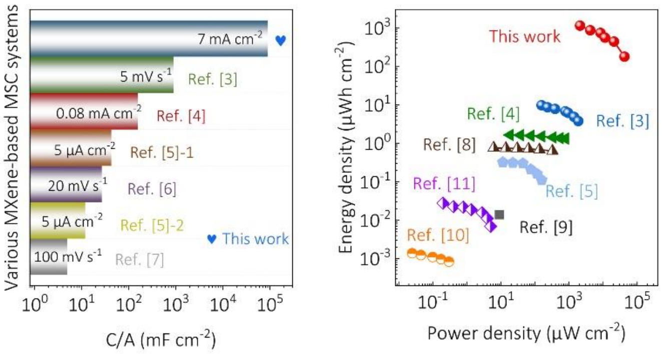

Supplementary Figure 30. a) Areal capacitance (C/A) comparison of this work to other reported MXene-based MSC systems, where Ref. [3] is extrusion-printed MXene,[3] Ref. [4] is screen-printed MXene sediment ink,[4] Ref. [5]-1 is extrusion-printed MXene,[5] Ref. [6] is spray-coated MXene,[6] Ref. [5]-2 is inkjet-printed MXene [5] and Ref. [7] is pen-written MXene.[7] **b**) Ragone plot comparison of this work to other MSC systems, where Ref. [3] is extrusion-printed MXene,[3] Ref. [4] is screen-printed MXene sediment ink,[4] Ref. [5] is extrusion-printed MXene,[5] Ref [8] is stamped MXene,[8] Ref [9] reduced graphene oxide,[9] Ref. [10] is inkjet-printed graphene,[10] Ref. [11] is spray-coated graphene.[11]

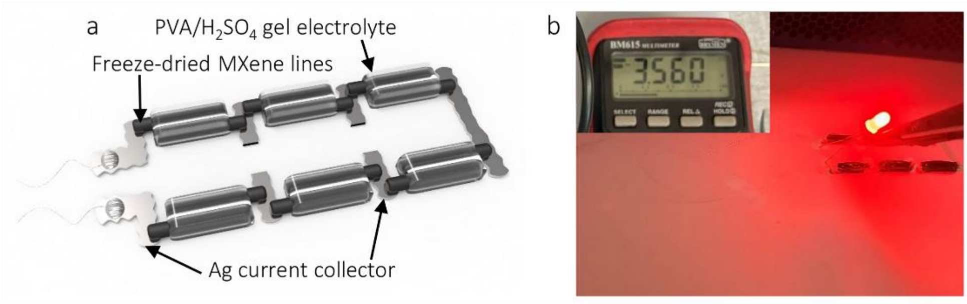

Supplementary Figure 31. a) Scheme of a MXene dough-based interdigitated tandem device and **b**) digital photograph of powering a LED after the device was charged to 3.6 V.

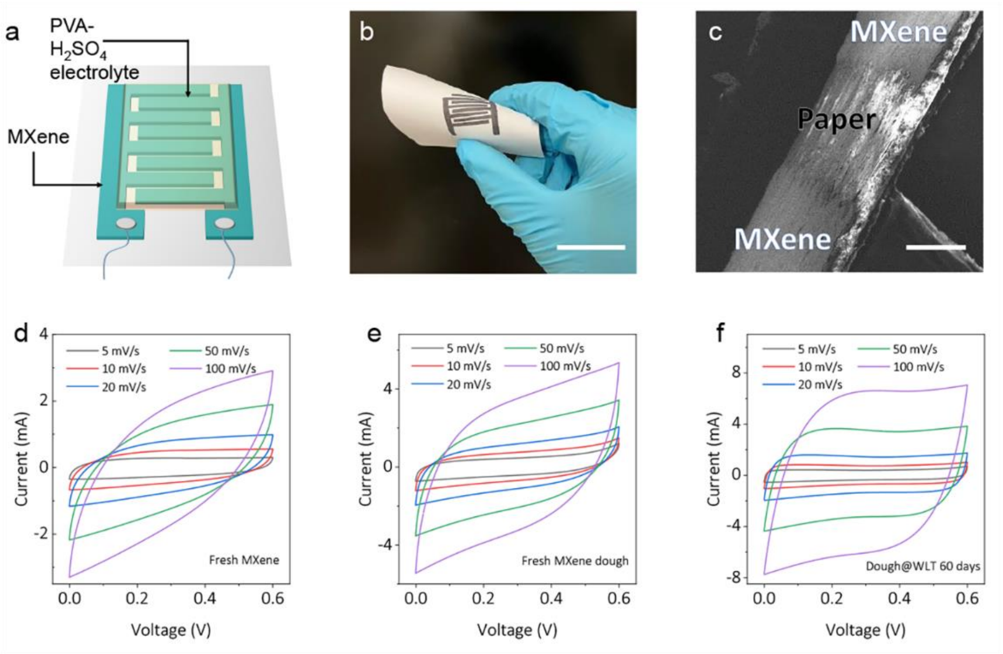

Supplementary Figure 32. **a**) Scheme and **b**) photo of as painted micro-supercapacitor (gap is 500 μm) from MXene dough. The scale bar is 3 cm. **c**) SEM of electrodes of the painted micro-supercapacitor, the scale bar is 300 μm. CV profile of micro-supercapacitors based on **d**) fresh MXene, **e**) fresh MXene dough and **f**) Dough@WLT stored over 60 days.

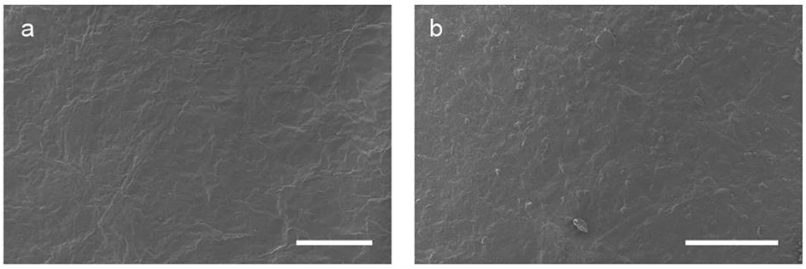

Supplementary Figure 33. SEM of the morphology of MXene electrode surface based on **a**) fresh MXene and **b**) Dough@WLT stored over 60 days. The scale bars are 200 μm. The rough surface of the latter with small MXene particles and slight oxidation contributes to higher charge storage performance.

Reference

[1] K. Maleski, C. E. Ren, M. Q. Zhao, B. Anasori, Y. Gogotsi, *Acs Applied Materials & Interfaces* **2018**, 10, 24491.

[2] P. W. Winston, D. H. Bates, *Ecology* **1960**, 41, 232.

[3] Y. Z. Shao, L. S. Wei, X. Y. Wu, C. M. Jiang, Y. Yao, B. Peng, H. Chen, J. T. Huangfu, Y. B. Ying, C. Zhang, J. F. Ping, *Nature Communications* **2022**, 13, 3223.

[4] S. Abdolhosseinzadeh, R. Schneider, A. Verma, J. Heier, F. Nuesch, C. F. Zhang, *Advanced Materials* **2020**, 32, 2000716.

[5] C. F. Zhang, L. McKeon, M. P. Kremer, S. H. Park, O. Ronan, A. Seral-Ascaso, S. Barwich, C. O. Coileain, N. McEvoy, H. C. Nerl, B. Anasori, J. N. Coleman, Y. Gogotsi, V. Nicolosi, *Nature Communications* **2019**, 10, 1795.

[6] Y. Y. Peng, B. Akuzum, N. Kurra, M. Q. Zhao, M. Alhabe, B. Anasori, E. C. Kumbur, H. N. Alshareef, M. D. Ger, Y. Gogotsi, *Energy & Environmental Science* **2016**, 9, 2847.

[7] E. Quain, T. S. Mathis, N. Kurra, K. Maleski, K. L. Van Aken, M. Alhabeb, H. N. Alshareef, Y. Gogotsi, *Advanced Materials Technologies* **2019**, 4, 1800256.

[8] C. F. Zhang, M. P. Kremer, A. Seral-Ascaso, S. H. Park, N. McEvoy, B. Anasori, Y. Gogotsi, V. Nicolosi, *Advanced Functional Materials* **2018**, 28, 1705506.

[9] J. J. Yoo, K. Balakrishnan, J. S. Huang, V. Meunier, B. G. Sumpter, A. Srivastava, M. Conway, A. L. M. Reddy, J. Yu, R. Vajtai, P. M. Ajayan, *Nano Letters* **2011**, 11, 1423.

[10] S. S. Delekta, A. D. Smith, J. T. Li, M. Ostling, *Nanoscale* **2017**, 9, 6998.

[11] N. Kurra, B. Ahmed, Y. Gogotsi, H. N. Alshareef, *Advanced Energy Materials* **2016**, 6, 1601372.
